# Composite and Nanocomposite Metal Foams

**DOI:** 10.3390/ma9020079

**Published:** 2016-01-28

**Authors:** Isabel Duarte, José M. F. Ferreira

**Affiliations:** 1 Department of Mechanical Engineering, TEMA, University of Aveiro, Campus Universitário de Santiago, Aveiro 3810-193, Portugal; 2 Department of Materials and Ceramics Engineering, CICECO, University of Aveiro, Campus Universitário de Santiago, Aveiro 3810-193, Portugal; jmf@ua.pt

**Keywords:** metal foams, composite metal foams, metal matrix composites (MMC) foams, micro and nano reinforcements, ceramic particles, carbon nanotubes, hollow spheres

## Abstract

Open-cell and closed-cell metal foams have been reinforced with different kinds of micro- and nano-sized reinforcements to enhance their mechanical properties of the metallic matrix. The idea behind this is that the reinforcement will strengthen the matrix of the cell edges and cell walls and provide high strength and stiffness. This manuscript provides an updated overview of the different manufacturing processes of composite and nanocomposite metal foams.

## 1. Introduction

The non-flammable, recyclable and lightweight open-cell and closed-cell metal foams have been used as functional and structural engineering applications [1,2,3,4]. The open-cell metallic foams are extensively utilized as heat exchangers, filters, electrodes, shock absorbers taking advantages of their high specific surface area and their high thermal and electrical conductivities [1,2]. The closed-cell metal foams, in particular the aluminum alloy (Al-alloy) foams, have been used in structural engineering applications (e.g., automotive, aerospace, industrial equipment and building construction) that require lightweight structures with high strength-to-weight and stiffness-to-weight ratios, high impact energy absorbing capacity and/or with a good damping of noise and vibration [3,4]. These foams, in particular the closed-cell aluminum foams, are usually applied as core and/or as filler of sandwich panels [5] and thin-walled structures [6], respectively. In these composite structures, the thin metal sheets [7] and the thin-walled structures (e.g., empty tubes) [8,9] ensure the high mechanical strength, while the foam core or filler mainly contribute to the high crashworthiness [6,10]. Although the relative low mechanical strength of these foams does not limit its range of applications, research efforts have been made towards enhancing the mechanical performance of the existing foams or/and developing high-strength Al-alloy foams or even a new class of high-strength foams in order to broaden the range of the applications of these materials. This could be achieved by strengthening the metal cell skeleton (metal-matrix) or/and by optimizing the pore structures. The most cost-effective ways currently used to enhance the mechanical properties of the existing Al-alloy foams are: (i) selecting a strong Al-alloy (in form of powder or ingot); or (ii) applying conventional heat treatment processes (e.g., age precipitation and annealing) usually used for Al-alloys [11,12,13,14]. Heat treatments promote the formation of precipitates from solid solution (precipitation hardening or age hardening) or the diffusion of alloying element into the matrix, forming a solid solution. Some authors studied the effects of these heat treatments on the mechanical properties for both open-cell [11,12] and closed-cell aluminum foams [13,14]. Zhou *et al.* [11] investigated the effects of annealing and T6-strengthening treatments on the compressive deformation behavior of open-cell aluminum foams (10 PPI) and found that the T6-strengthneing treatment increases the compressive strength of these foams (e.g., the peak stress changed from 2.2 to 3.2 MPa). On the other hand, the thermal annealing has negative effects on the compressive strength of these foams, decreased the main mechanical parameters (the peak stress decreased from 2.2 to 1.0 MPa, after the treatment). Yamada *et al.* [12] found a similar effect of SG91A (Al-9 wt.% Si-0.5 wt.% Mg-0.5 wt.% Fe-0.4 wt.% Mn) and AZ91 Mg (Mg9 wt.% Al1 wt.% Zn-0.2 wt.% Mn) alloys. Similar conclusions were drawn for closed-cell aluminum alloy foams. For example, the compressive strength of 6101 [13,14] and 7075 [13] Al-alloy foams could be improved by applying a suitable T6-strengthneing treatment. Nonetheless, some of these heat treatments develop cracks in the cell walls due to the thermal stresses that are responsible for the oscillation of the stress in the plateau region [14]. The mechanical performance of the existing foams could be also increased by diminishing the size of their cellular pores, demonstrated by Xia *et al.* [15] and by Jiang *et al.* [16]. Nonetheless, these aforementioned methodologies allow slightly increasing the mechanical strength of such foams, but not allow the fabrication of high strength foams.

Recently, great efforts have been carried out to fabricate the high strength metal foams. Most of the ideas have emerged based on the research which has been carried out to fabricate high-strength solid metals [17,18]. One strategy for strengthening of the Al-alloys is adding alloying elements (e.g., Mg, Ni) to promote the formation of intermetallics (e.g., precipitation hardening) [17]. Another strategy is incorporating micro and nano-sized reinforcement elements into the metal bulk matrix to enhance the performance of the ductile metal [18]. For example, ceramic particles, e.g., alumina (Al_2_O_3_), silicon carbide (SiC) [17], ceramic fibers [17], ceramic nanoparticles [19] are some of the most attractive reinforcing materials. More recently, there has been a crescent interest in exploring carbonaceous materials as reinforcing agents for metal alloys [20,21,22]. This became an attractive research field both from the scientific and industrial applications viewpoints. Considerably research activities involving the metal matrix nanocomposites have been undertaken. Till to present, research efforts have been mainly concentrated on Al-alloys reinforced with carbon nanotubes [20,22]. As it is well-known, carbon nanotubes (CNTs) have high aspect ratio (*i.e.*, length to diameter, or length to thickness ratio) and exhibit an amazing high elastic modulus and mechanical strength, as well as, excellent electrical and thermal conductivities [21]. As a result, they are considered to be the most effective reinforcing elements for fabricating composite materials for structural and functional engineering applications [23]. Although the results are promising, the incorporation of CNTs in metallic matrices is still an unsolved problem due to their high tendency to agglomerate into clusters, their poor dispersion ability in the metal-matrix, and the poor wettability of carbon by molten metal. The poor wettability derives from the large difference in surface tensions between CNTs and molten metal. The formation of interfacial reaction products leading to loss of their structural integrity is another drawback. These are the current challenges that need to be overcome in this metal matrix composite field [20]. These reinforced materials with micro- and nano-sized reinforcing elements are designated as metal matrix composites (MMC) or metal matrix nanocomposites, respectively.

This paper presents an overview of the main strategies that emerged recently aiming at fabricating composite and nanocomposite metal foams with enhanced mechanical performance, with an especial focus on Al-alloy foams that are the most industrially used ones. This review focuses processing and the properties, as well as the strengthening mechanisms associated for the reinforced foams. The reinforced foams are compared to the conventional open-cell foams and closed-cell foams.

## 2. Composite Metal Foams

### 2.1. Metal Foams Reinforced with Ceramic Particles

The micro-sized ceramic particles were firstly used in the metal foams field to promote the liquid foam stability and avoid the formation of non-uniform pore sizes. The ceramic particles migrate preferentially to the liquid/gas interface and stabilize the bubbles, while increase the viscosity of the melt [24]. Ceramic particles prevent drainage of metallic melt and the coalescence of bubbles, which are common causes of non-uniform structures in foams. The preferential migration of the particles to the liquid/gas interfaces is mostly related to the differences in surface tensions between the solid particles and the molten metal and the poor wetting ability. In this regard, ceramic particles in metal foams play a stabilizing role similar to that of surfactants in many other foam systems (emulsions, aqueous liquid foams, *etc.*). The presence of ceramic particles at the liquid/gas interfaces contribute to the formation of bridges between opposite liquid/gas interface, preventing them to get closer, burst the bubbles and promote their coalescence. Accordingly, they tend to retard the flow of liquid out of the foam and to hinder the growth of the bubbles, contributing to a more uniform foam structure.

Ceramic stabilizing particles (e.g., SiC and Al_2_O_3_) are required to fabricate closed-cell Al-alloy foams by direct foaming technique, which is one of the most common and economical methods [24,25]. The ceramic particles migrate preferentially to the liquid/gas interfaces and stabilize the bubbles, while increase the viscosity of the melt [25]. Ceramic particles prevent drainage of metallic melt and the coalescence of bubbles, which are common causes of non-uniform structures in foams. Direct foaming methods start from a molten metal containing dispersed ceramic particles, into which gas bubbles are injected directly [26], or generated chemically by the decomposition of a blowing agent (e.g., titanium hydride, calcium carbonate), or by precipitation of gas dissolved in the melt by controlling temperature and pressure [27]. When dispersed into the molten metal, ceramic particles adhere to the gas/metal interfaces of rising bubbles, avoiding their burst [25]. However, high volume fractions of ceramic particles (above 10 vol.%) are usually required to effectively the foams. For example, AlSi7Mg (A356) foams are prepared by dispersing 20 vol.% SiC particles (SiCp) in the AlSi7Mg melt containing titanium hydride as blowing agent [28]. Accordingly, the resulting reinforced foams exhibit a brittle mechanical behavior conferred by such high particle contents, preferentially located in the cell-wall where they induce localized deformations. This is the main reason why such lightweight reinforced foams are not recommended for structural applications that require a ductile behavior. This type of foam is commonly used in building construction fulfilling other roles, such as acoustic and thermal insulation [2,3]. Furthermore, the high volume fraction of ceramic particles in these foams make cutting and machining difficult (increasing of the machining time) contributing to an increase of the production costs. Reducing the size of reinforcing particles leads to a higher number of particles for a given content, and might allow decreasing the volume fraction required for an effective foam stabilization, with concomitant reductions of costs and brittleness of the foams.

Ceramic particles have been explored also to control the cellular structure (e.g., size of the cellular pores) minimizing the structural defects and imperfections to improve the quality of foams as detailed elsewhere [29]. Exploring the role of ceramic particles as reinforcing materials was also undertaken as a route to improve the mechanical properties of the metal matrix of the existing foams. In the reality, all these features are closely interrelated to each other since the cellular structure (e.g., pore size) and density gradients strongly influence the mechanical properties of the resulting metal matrix composite foams. Various micro-sized ceramic particles have been explored as both stabilizer and reinforcing elements, including Al_2_O_3_ [30,31,32], SiC [33,34,35,36,37,38,39,40,41], titanium diboride (TiB_2_) [42], yttrium oxide (Y_2_O_3_) [43] and AlN [44]. Ceramic particles can be directly dispersed in the molten Al-alloy or be *in-situ* formed through chemical (e.g., oxidation, formation of oxide bi-films) and metallurgical reactions (e.g., intermetallic compounds). The extent of the *in-situ* formed particles can be tailored by controlling the atmosphere and the agitation promoted by the rise of injected gas bubbles, or by applying further mechanical stirring. Adding oxygen seeking elements of the Group 2 (beryllium, magnesium, calcium, strontium and barium) is a common way to foster internal oxidation. For example, stable Alporas foams are prepared by adding calcium to an aluminum melt under mechanical stirring and air injection enhance the viscosity of the melt (thickening) due to the *in-situ* formation of oxide particles. A blowing agent is then added into the melt. Another approach for the *in-situ* formation of stabilizing sub-micrometer carbide and boride particles in metal foams consisted in adapting the flux assisted melting method [45] already known in master alloys containing grain refiners. For example, composites consist of Al-alloys containing ceramic particles of controlled size (e.g., TiB_2_, TiC) were prepared by this method using fluoride salts [45].

The findings reported in a number of published work involving different manufacturing methods are summarized in Table 1 and Table 2. Direct and indirect foaming methods have been used, in which the micro-sized particles are dispersed directly into the molten metal at high temperatures under mechanical stirring [24]; or previously mixed with metal powders based on powder metallurgy (PM) method [46], respectively. PM method [47,48], one of the most commercially exploited to fabricate the closed-cell metal foams, consists on heating of a precursor material obtained by hot compaction of a metal alloy (e.g., Al-alloy) with blowing agent powders (e.g., titanium hydride, TiH_2_). Under the internal gas pressure derived from the decomposition of the blowing agent uniformly dispersed in the precursor, the metal expands and acquires a porous structure of closed-cells. A good coincidence should exist between the thermal decomposition of the blowing agent with the release of a gas (e.g., hydrogen, H_2_) and the melting of the metal [48]. The liquid foam is then solidified by cooling in air to obtain solid foams consisting of closed cells, and covered by a thin external dense metal skin conferring them a good surface finish [49]. This enables producing foams with porosities between 75% and 90% [48]. The ability of PM method to produce metal foam components with different architectures (e.g., sandwich systems, filled profiles and 3D complex shaped structures) is its main advantage [49]. Furthermore, different materials or structures can be joined during the foaming step, without using chemical adhesives. For example, fasteners or standard parts used in vehicles (*i.e.*, nuts, bolts, screws, pin rivets) could be incorporated into the metallic foam during its formation [50]. PM method also allows fabricating *in-situ* foam-filled tubes in which Al foam fills empty Al-alloy tubes while expanding during the foaming process [6]. High production costs derived mostly from the precursor preparation, and the difficulty to fabricate large volume foam parts are the principal disadvantages of the PM process. Herein, the micro-sized ceramic particles (inferior to 10 vol.%) are directly dispersed in the powder mixture of metal and blowing agent using mechanical mixers (e.g., turbula). Dense reinforced precursors are obtained by hot compacting the powder mixture. PM method requires lower volume fractions of ceramic particles in comparison to direct melt foaming.

An effective dispersion of particle reinforcements in the matrix it is essential for taking full advantages of their incorporation. However, micro-sized particles are often found not well-dispersed into the metallic matrix. Their tend to aggregate and agglomerate in clusters that appear in the cell-walls has been attributed to surface tension effects, but less attention has been given to the pre-existing agglomerates in the starting ceramic powders. The easiness how the micro-sized particles react with the metal-matrix during melting and foaming to form intermetallic products that increase the viscosity of the melt is another drawback. Thickening and foam stabilization mechanisms can be assessed by quantifying the phases formed during the entire melt foaming process [51,52,53]. Phase analysis is also expected to shed light on foam fracture behavior [54,55]. However, there the impact of phase changes on the foaming process is not consensual. Some authors claiming that intermetallic phases are important, while others holding that oxides are the main responsible for foam stability [56]. Moreover, the gas released from blowing agents (e.g., titanium hydride and calcium carbonate) can accumulate at the interface between these reinforcement particles and metal matrix during the foaming process, leading to a weak interfacial bonding. This inevitably limits their reinforcing potential.

The findings summarized Table 1 and Table 2 reveal that besides the stabilizing role of ceramic particles present in metal matrix composite (MMC) foams, they also increase the stiffness and the fragile behavior of the foams. Therefore, for a given system (Al-alloy and stabilizing/reinforcing ceramic particles) the experimental variables (type of particles, mean size, content of particles, pore volume fraction, *etc.*) have to be compromised taking into account the overall changes induced in the manufacturing process of MMC foams.

**Table 1:** Literature survey on ceramic particles reinforced foams prepared by powder metallurgy.

Reference	Metal	Ceramic Particle	Manufacturing	Test Conditions	Conclusions
Elbir *et al.* [33]	Al	SiC 8.6–20 vol.% Size: 22 μm	Powder Metallurgy Al-powder: <74 μm TiH_2_-powder: <37 μm	Φ 20 mm × 20 mm Compression: Quasi-static 0.1 mm·s^−1^	In comparison to non-reinforced Al foams, SiCp particles reduce the drainage and cell coarsening phenomena, increase linear expansion and compressive strength of Al foams, but induce fluctuations in the plateau region of stress-strain curves and accentuate the brittle behavior of composite foams.
Esmaeelzadeh *et al.* [34]	AlSi7	SiC up to 10 vol.% Size: 3–16 μm	Powder metallurgy Al powder: <160 μm Si powder: <150 μm TiH_2_-powder: <63 μm	Φ 30 mm × 40 mm Compression: Quasi-static 1.1 × 10^−3^ s^−1^	Increasing the added amounts of SiCp or decreasing their size reduce the drainage but lead to less homogeneous foam structures. The compressive properties and energy absorption efficiency are degraded due to an accentuation of brittleness in comparison to non-reinforced AlSi7 foams.
Kennedy and Asavavisitchai [42]	Al	TiB_2_ Size: 10 μm 6 vol.%	Powder metallurgy Al powder: 48 μm TiH_2_ powder: 33 μm	Φ 22 mm × 24 mm Compression: Quasi-static 0.5 mm·min^−1^	TiB_2_ particles significantly enhance the maximum foam expansion but did improve the long-term stability of the foams due to their poor wetting by the molten Al, as evidenced by particles protruding the cell-walls into the gas phase. The stress-strain curves in plateau region are smooth and characterized by a slightly increasing slope, irrespective of the presence or the absence of reinforcement. The maximum yield stress is achieved for TiB_2_-Al composite foams.
Guden and Yuksel [35]	Al	SiC 0–20 vol.% Size: 22 μm	Powder metallurgy Al powder: 34.64 μm TiH_2_ powder: <37 μm	Φ 13 mm × 13 mm Compression: Quasi-static 3 × 10^−3^ s^−1^	SiCp increase the linear foam expansion by increasing the bulk viscosities. The composite SiCp-Al foams are more brittle in comparison to with Al-foams.
Alizadeh and Mirzaei-Aliabadi [30]	Al	Al_2_O_3_ Size: 10 μm 0–10 vol.%	Space-holder Al-powder Carbamide: 1.2 mm Ethanol: 1–3 wt.%	Φ 25 mm × 30 mm Compression Quasi-static 0.1 mm·s^−1^	Increasing volume fractions of Al_2_O_3_p enhance the Young’s modulus and the compressive strength of the composite foams in extends that depend on the porosity fraction. For a given porosity fraction, the plateau region of composite foams is less smooth and shorter than for the Al-foam. The plateau stress and energy absorption capacity increase with Al_2_O_3_p content increasing up to 2 vol.%, but this trend is reversed for higher volume fractions. However, contrarily to other literature reports [34,41], the energy absorption efficiency of the composite foams is always higher than that of non-reinforced Al-foams.
Luo *et al.* [36]	AlSi9Mg	SiC 4 vol.% Size: 28 μm	Infiltration process AlSi9 alloy NaCl (0.9–4 mm in size)	15 mm × 15 mm × 35 mm Compression Quasi-static 10^−3^ s^−1^	SiCp increase yield stress and energy absorption capacity of composite foams increase. Stress-strain curves of composite foams are less smooth than as than those of non-reinforced foams.
Zhao *et al.* [43]	Al	Y_2_O_3_ 0.3–1.2 wt.% Size: 50 μm	Space holder Al powder NaCl particles: 0.66–0.90 mm	12.8 mm × 6.5 mm × 35 mm Compression Quasi-static 3 mm·min^−1^	Volume fractions of Y_2_O_3_p up to 0.8 wt.% enhance bending strength up to a maximum of 20.4 MPa, a trend that is reversed for further added amounts, while the maximum micro hardness is achieved within the range of 0.5–0.8 wt.%.

**Table 2:** Literature survey on ceramic particles reinforced foams prepared by direct foaming methods.

Reference	Metal	Ceramic Particle	Manufacturing	Test Conditions	Conclusions
Liu *et al.* [41]	Zn-22Al	SiC Size: 28 μm 7 vol.%	Direct melt foaming ZA22 alloy ingot CaCO_3_: 44 μm	15 mm × 15 mm × 30 mm Compression Quasi-static (2.2 × 10^−3^ s^−1^)	SiCp accentuate the brittleness and enhanced the stress fluctuations within the plateau region of composite foams. The energy absorption capacity is slightly improved but the energy absorption efficiency is degraded in comparison to non-reinforced foams.
Luo *et al.* [37]	AlSi9Mg	SiC Size: 28 μm 0–20 vol.%	Direct melt foaming AlSi9Mg alloy CaCO_3_: 44 μm	15 mm × 15 mm × 35 mm Compression Quasi-static (10^−3^ s^−1^)	The same conclusions as above [41]. At a given relative density, yield and collapsing stresses of composite foams increase with increasing SiCp volume fraction.
Yu *et al.* [38]	Zn-22Al	SiC Size: 28 μm 10 vol.%	Direct melt foaming ZA22-powder: 40 μm CaCO_3_: 44 μm	15 mm × 15 mm × 30 mm Compression Quasi-static (2.2 × 10^−3^ s^−1^) Φ 70 mm × 10 mm Damping (400 Hz)	The same conclusions as above [37,41]. The damping capacity of composite foams is slightly higher than those of ZA22 alloy and ZA22 foams.
Yu et al [39]	AlSi9Mg	SiC Size: 28 μm 10 vol.%	Direct melt foaming AlSi9Mg alloy CaCO_3_: 44 μm	15 mm × 15 mm × 35 mm Compression Quasi-static 5 × 10^−4^–1 × 10^−2^ s^−1^ Φ 30 mm × 10 mm High strain rate (600; 1600 s^−1^)	The same conclusions as above concerning the effects of SiCp on the mechanical properties of composite foams [37,38,41]. The yield stress depends on both relative density and strain rate, being 10 MPa and 40 MPa for quasi static (<10^−2^ s^−1^) and dynamic (1600 s^−1^) loading conditions.
Dauod [32]	A359	Al_2_O_3_ 0–15 vol.% Size: 50–140 μm	Direct foaming CaCO_3_	Compression Quasi-static 3 × 10^−3^ s^−1^	Al_2_O_3_p enhance the uniformity of foam microstructure and the resulting compressive stress-strain curves of composite foams are smooth. The mechanical parameters increase almost linearly with increasing the volume fraction of Al_2_O_3_p. The energy absorbing capacity is not much sensitive to the volume fraction of Al_2_O_3_p up to 10 vol.%, increasing for higher contents.
Song *et al.* [44]	Al-3.7 Pct Si-0.18 Pct Mg	AlN	Solid/liquid reaction Master ingot	10 mm × 10 mm × 10 mm Compression Quasi-static (1 mm·min^−1^)	AlNp reveal an effective reinforcing role increasing the mechanical properties of Al-alloy foams. Absence of stress oscillations in the plateau region of strain-stress curves of composite foams, similarly as observed for Al_2_O_3_p [32] and TiB_2_p [42] Al composite foams.

However, detailed evolution trends of foams’ properties as a function of a given experimental variable can hardly be drawn because of the diverse manufacturing and testing conditions used in different studies. More straightforward conclusions would require a systematic experimental approach under strictly controlled conditions, namely concerning the influence of particle size on foaming behavior and mechanical properties. In an attempt to illustrate the findings on this topic reported by Esmaeelzadeh *et al.* [34], Table 3 summarizes the data estimated from the plots published in this single work on the subject. SiC particles of different size were used to reinforce AlSi7 foams prepared by PM method, using TiH_2_ as blowing agent. It can be seen that all the mechanical parameters are favored when smaller particles are used.

**Table 3:** Effect of the particle size and volume fraction of SiC particles on the compressive behavior of AlSi7 foams [34].

SiC Size	SiC (vol.%)	Yield Stress (MPa)	σ_0.1_ (MPa)	σ_0.2_ (MPa)	σ_0.3_ (MPa)	σ_0.4_ (MPa)
3 μm	0	1.13	1.13	1.38	1.41	1.50
	3	1.58	1.33	1.72	1.75	1.88
	6	1.25	1.13	1.33	1.41	1.88
16 μm	3	1.25	1.0	1.25	1.38	1.58

**Figure 1:**
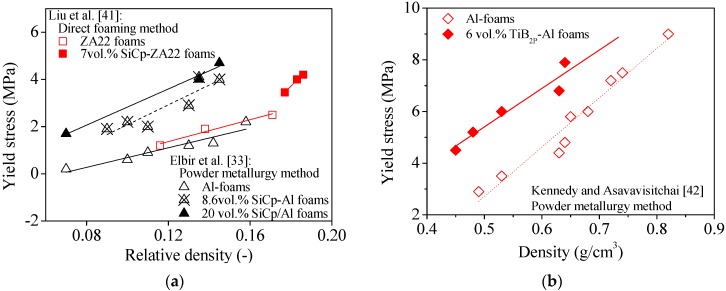
Effect of the volume fraction of SiC (**a**) and TiB_2_ (**b**) particles on the yield stress of Al based foams.

**Figure 2:**
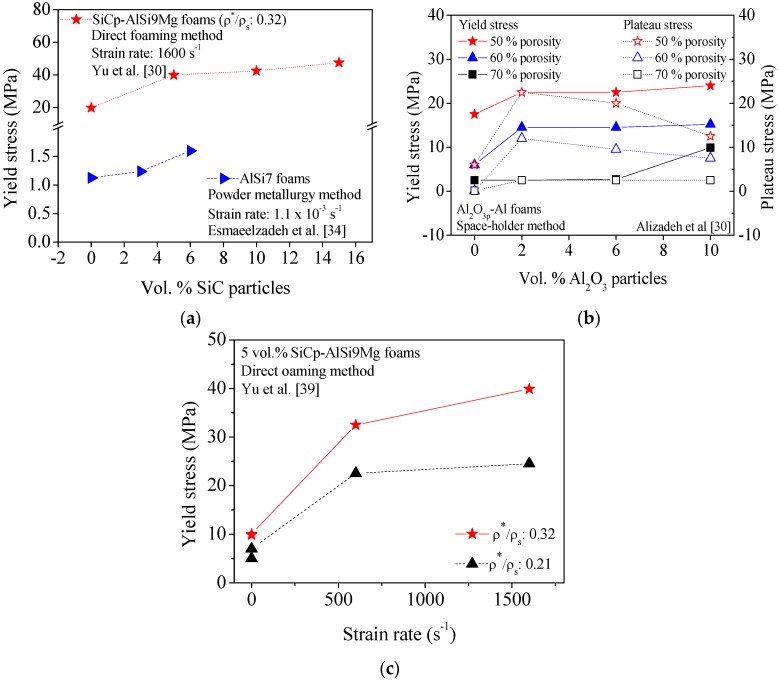
Effect of the volume fraction of particles on the yield stress (**a**,**b**) and plateau stress (**b**) of foams containing SiC (SiCp) (**a**), and Al_2_O_3_ particles (Al_2_O_3_p) (**b**). Effect of strain rate on compressive behavior of reinforced foams containing 5 vol.% SiC particles is displayed in (**c**).

In an attempt to sort out some general trends from previous studies, Figure 1 and Figure 2 condense some published data about on the effects of micro-sized ceramic particles (SiCp, TiB2_p_, and Al_2_O_3p_) on the compression behavior of different metal (ZA22, Al, AlSi9Mg and AlSi7) foams produced through different manufacturing processes. The plotted data reveal that, irrespective of the foaming method and metal matrix, the yield stress of composite foams is always higher in comparison to non-reinforced ones (Figure 1 and Figure 2). The yield stress also shows a general increasing trend with increasing contents of ceramic particles as shown in Figure 1a and Figure 2a,b and Table 1 and Table 2. However, the plateau stress strongly depends on the porosity fractions, as expected.

**Figure 3:**
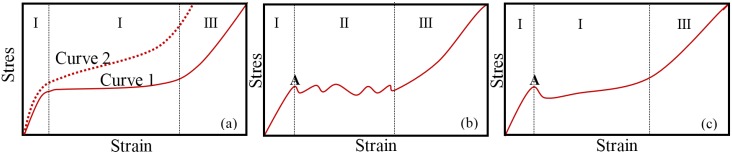
Schematic representation of typical stress-strain curves (**a**–**c**) measured for metal foam under uniaxial compression characterized by quasi-static (I); plateau (II); and densification (III) regions.

Using stronger and stiffer reinforcing ceramic particles also enhance the cell walls strength. Figure 3 shows a schematic representation of typical compressive stress-strain curves often measured for metal foams under uniaxial compression. These curves usually comprise three regions (quasi-elastic, plateau and densification). Region I extends from the beginning up to the yield stress; the plateau region (II) characterized by a flat and smooth stress plateau (Curve 1, Figure 3a), sometimes with slight increasing slope (Curve 2, Figure 3a), or presenting some fluctuations (Figure 3b); and finally the densification (III) region. Furthermore, it is often difficult to precisely define the yield point (Point A in Figure 3b,c) as the curves shown in Figure 3a.

From the data published up to the present, the stress-strain curves of composite foams usually exhibit stress fluctuations within the plateau region (Figure 3b), irrespective of the manufacturing process. However, in the particular cases of foams reinforced with TiB_2_p [42] produced by the PM method (Table 1) and composite foams reinforced with Al_2_O_3_p [32] and AlNp [44] prepared based on direct foaming method (Table 2), the absence of stress oscillations in the plateau region of strain-stress curves were found (Figure 3a,b).

### 2.2. Metal Foams Reinforced with Intermetallics

Stronger high-strength Al-alloy powders or ingots can be directly used for preparing high-strength Al-alloy foams. In comparison to pure Al-foams, AlZn5Mg1, AlSc0.24 [57,58], AlZn7Mg0.5 [59], AlZn10Mg0.3 [59,60] enabled stronger foams to be formed without compromising the foaming process. This makes alloying methodologies promising for the fabrication of reinforced Al-alloy foams with high strength. Alloying elements are directly added to the melt or dispersed in the powder mixture through direct and indirect melt foaming methods, respectively. Magnesium (Mg), manganese (Mn), scandium (Sc), nickel (Ni), and cobalt (Co) are some of the most studied alloying elements aiming at enhancing the mechanical properties of the existing foams [61,62,63,64,65,66,67,68,69,70,71]. The role of alloying elements on the foaming behavior of the Al-alloys, namely through the thickening and foam stabilization mechanism has also been investigated. The phases formed during the foaming melt process and the phases remaining in the solidified foams have been extensively studied due to their impact on fracture of the resulting foams [54,55]. Several strengthening mechanisms have been postulated. Some authors claim that the intermetallic phases formed are essential for foam stabilization, but others defend that formed oxides play here a more important role.

The replacement of calcium in the direct foaming method [69,70] by magnesium (Mg) as foam stabilizer was found to promote a non-uniform pore-size distribution with increasing added amounts of Mg with the resulting cell size gradient foams. This negative effect on the efficacy of metallic foam strengthening [59] might be attributed to the higher affinity of Mg towards oxygen in comparison to that of calcium. The higher affinity of Mg towards oxygen favors the formation of MgO or MgO-containing phases such as spinel [72], which, in turn might cause foaming problems.

**Figure 4:**
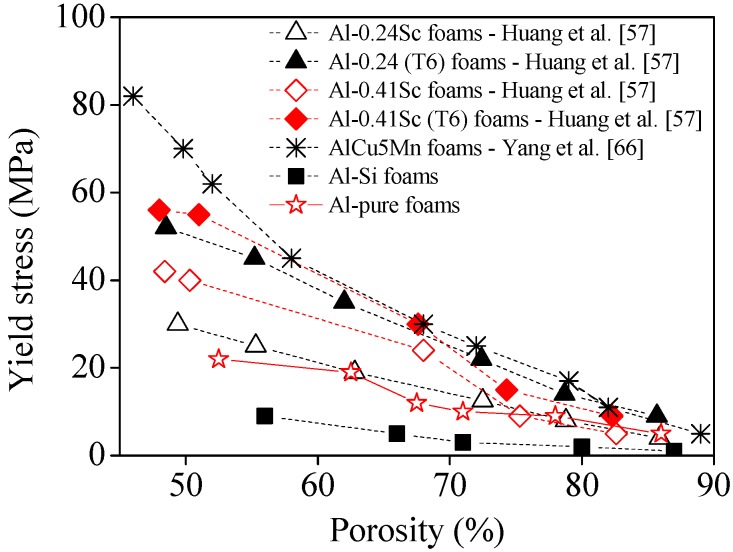
Comparison of different Al-based foams [57,66].

Huang *et al.* [57] studied the effect of scandium on the quasi-static compressive properties of Al-alloy foams. They found that the addition of small volume fraction of scandium coupled with an appropriate heat treatment of the resulting composite solid foam, improved their compressive yield strength (Figure 4), which was attributed to the formation of precipitates (e.g., Al_3_Sc). The effect tended to increase with increasing volume fractions of Sc, and with post heat treatment, as shown in Figure 4.

The stress-strain curves are smooth and, as expected, lower stresses are required for deformation with increasing porosity, and the plateau are shorter and more inclined as density increases. Moreover, the benefits of the post heat treatment that promotes a more extensive precipitation of intermetallic phase, are obvious when comparing with the heat treated (b,d) with non-treated (a,c) foams. The formation of Al3Sc precipitates at the grain boundaries of Al-Sc alloy foams was well evidenced by TEM images, which also showed atomic dislocations in <001> Al projection.

The authors also compared the Sc-containing foams with other intermetallic-reinforced foams, as well as with pure Al-foams. Except for the conventional Al-Si foams, the results (Figure 4) confirmed the superior mechanical properties of the other intermetallic-reinforced foams. Interestingly, the AlCu5Mn revealed to be generally stronger than the Sc-containing ones even after post heat treatment. Considering that Sc-containing foams are very expensive, the AlCu5Mn including cheaper metals appear as very competitive.

Manganese (Mn) was also shown to be an ideal element to promote reinforcement through the formation of intermetallics, enhancing micro hardness, yield stress and the plateau region of the stress-strain compressive curves of the Al-alloy foams [66]. The Mn volume fraction should be adjusted in order stabilize a good compromise between the yield strength and ductile deformation behavior [66]. The benefits of adding Mn contents ≥4 wt.% are not clear. Intermetallics-reinforced close foams were prepared by the melt foaming method using Ca as stabilizer and TiH_2_ as blowing agent, varying the volume fraction of Mn powder (100 mesh). Mn was found uniformly distributed in the matrix as oxides (e.g., MnO_2_), intermetallics (e.g., Al6Mn), Al-Mn solid solution (solid solution strengthening), as well as Mn particles non-dissolved in the cell wall matrix. The resulting reinforced foams possessed a much higher energy absorption capability compared to the Al-foams, which increased with increasing added amounts of Mn. It can be seen that the plateau appears gradually more inclined with increasing Mn contents.

The effects of Mn contents on mechanical properties of Mn-containing Al-foams suggest that compressive yield strength is more sensitive to the presence of Mn than micro hardness. This is likely to be related to the amount of oxides formed in the Al-Mn matrix.

Aguirre-Perales *et al.* [67] performed a systematic study on the effect of the different alloying elements (Co, Mg, Mn, Ni and Ti) on the compression behavior and the energy absorption capability of Al-3 wt.% Sn alloy. Their observations confirmed the general findings of the previous literature reports in the field that energy absorption of Al-foams and their contents in intermetallic phases increase with increasing contents of these alloying elements up to a certain limit.

In summary, the alloying elements usually enhance the mechanical properties of the metallic foams by favoring the formation of intermetallic compounds by reacting with oxygen from the atmosphere.

### 2.3. Composite Foams Reinforced with Hollow Spheres

**Figure 5:**
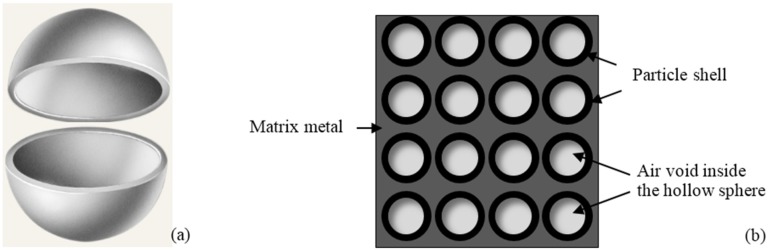
Scheme of a hollow particle (**a**); and its distribution on metal matrix of a syntactic foam (**b**).

Composite metallic foams incorporating lightweight filler particles such as hollow spheres are commonly designated as metallic syntactic foams (MSFs) and possess some particular features [73,74]. The term “Composite metal foam” was firstly introduced by Rabiei *et al.* [75] to designate a specific type of syntactic foams developed by PM and gravity casting techniques. The foams comprise steel hollow spheres packed into a random dense arrangement with interstitial spaces between spheres occupied with a solid metal matrix. These foams are fabricated, for example, by sintering a mixture of steel spheres and iron powder, or by infiltrating an aluminum alloy through the interstitial spaces between steel spheres. Figure 5 shows a schematic representation of an individual hollow sphere and of their arrangement in the composite syntactic foams.

The closed-cell structure is conferred by the hollow spaces instead of being derived from foaming. This allows easily controlling the cellular structure of composite foams. Furthermore, density and mechanical properties of MSFs can be easily adjusted by selecting the characteristics of both filler (e.g., volume fraction, size and wall thickness of the spheres) and the metal matrix (e.g., chemical composition). The aspect ratio, sphericity, density and chemical composition of the hollow spheres influence the mechanical behavior of the MSFs. This approach is also compatible with all the above referred strategies usually used to reinforce the alloy matrix, including the addition of micro- and nano-sized reinforcements, formation of intermetallics, oxides, thermal heat treatments, *etc*. MSFs are nowadays being considered as promising materials for commercial and military applications due to their very high level of energy absorption compared to the conventional materials.

Several alloys (e.g., magnesium [76,77,78], iron [77,78,79,80,81], steel [82,83,84,85,86,87,88,89,90], invar (FeNi_3_6) [89,90] and titanium [91,92] alloys have been tested as matrices of MSFS, although aluminum and its alloys [72,93,94,95,96,97,98,99,100,101,102,103,104,105,106,107,108,109,110,111] were the most studied. Hollow spheres of ceramics (e.g., Al_2_O_3_ [93] and SiC [94,95]), perlite particles [99,100,101], metals [82,88,111,112,113,114,115,116], glasses microspheres [79,81], pumice (low-cost natural volcanic glass) [117] and even of by-products such as fly ash cenospheres [76,78], are widely used as reinforcements in different sizes (from a few microns to several mm). The selection of the filler materials is based on criteria related to their mechanical properties, the compatibility with the metal matrix and the price. Table 4 and Table 5 summarize the results of a literature survey on MSFs based on Al-alloy, and iron or steel matrices, respectively.

Some ceramic hollow particles (e.g., SiC) can be easily wetted by molten alloys, while other need to be coated with different materials (e.g., nickel and copper) to enhance their compatibility with the metal matrix [73]. Optical micrographs of an Al-alloy (A356) reinforced with SiC hollow spheres show that the particles are wetted well with the alloy, even the closely spaced particles have a layer of matrix between them, wetting of particle with the matrix alloy and mechanical interlocking at the particle-matrix interface [94].

MSFs can be fabricated by the same conventional solidification processes (e.g., casting) and powder metallurgy methods [73]. In the solidification processes, the solid reinforcements are combined with the metal matrix in liquid state, casting and then solidified in a given desired shape. One common method is the pressure infiltration in which the liquid metal is forced to flow through the voids between the hollow spheres (HS). The pressure can be applied by an inert gas, vacuum infiltration, mechanical pressure. Another common method is the stir casting in which the hollow spheres are added in the molten metal under constant agitation and stirring. Herein, this method favors the segregation and the agglomeration of the hollow spheres. In the powder metallurgy processing, the solid reinforcements are previously mixed with metal powders, pressed into a shape, outgassed, and sintered by hot compaction for obtaining near full dense parts. The dense parts can undergo post heat treatments (forging, rolling and extrusion). A dispersing agent can be used to facilitate obtaining a homogeneous distribution of the hollow spheres in the metal powder.

**Table 4:** Literature survey on aluminum alloy syntactic foams.

Reference	Syntactic Foam Type	Testing Conditions	Results
Licitra *et al.* [93]	Matrix: A356 alloy Particles: Al_2_O_3_, 3 mm diameter and 105 μm wall thickness	Compression Quasi-static (10^−3^ s^−1^) High (445–910 s^−1^) Dynamic Mechanical properties	Young modulus, compressive strength and plateau stress of MSFs are directly proportional to density.
			Particle failure initiates the specimen failure, followed by shear failure of matrix and remaining particles.
			Storage modulus of A356 matrix and MSFs decreases but the loss modulus and damping parameter increase as temperature increases.
Cox *et al.* [95]	Matrix: A356 alloy Particles: SiC,1 mm diameter and 70 μm wall thickness	Compression Quasi-static (10^−3^ s^−1^) High (up to1520 s^−1^)	Evidences of hollow spheres crushing at the end of the elastic region.
			No strain rate sensitivity detected within the investigated range.
			Failure at high strain rate is initiated by particle cracking and shear band formation.
Balch *et al.* [96]	Matrix: cp-Al, 7075alloy Particles: crystalline mullite and amorphous silica hollow microspheres	Compression Quasi-static (10^−3^ s^−1^) High (up to2300 s^−1^)	Pure Al MSFs show compressive strength >100 MPa with a uniform densification plateau of 60% under quasi-static conditions.
			7075-Aluminum alloy exhibit significantly higher peak strength of up to 230 MPa under quasi-static conditions.
			HSR testing showed a 10%–30% increase in peak strength as com- pared to quasi-static testing and displayed energy absorbing capacity.
Orbulov *et al.* [97]	Matrix: Al99.5, AlSi12, AlMgSi1 and AlCu5 alloys Particles: ceramic hollow spheres with Al_2_O_3_, SiO_2_ and Mullite	Compression Quasi-static (free, 10^−2^ s^−1^) Quasi-static (Constrained, 10^−2^ s^−1^)	Densification limit was primarily influenced by the hollow spheres’ size in constrained compression.
			Recoverable energy in constrained compression case is influenced by the applied heat treatment.
			Overall absorbed mechanical energy is largely influenced by the compression mode (free or constrained).
Goel *et al.* [98]	Matrix: Al-2014 Particles: Aluminum cenospheres, 90 μm and 200 μm diameter	Compression Quasi-static (10^−3^ s^−1^) High strain (up to1400 s^−1^)	Syntactic foam shows about 10%–30% higher compressive strength under high strain rate conditions as compared to the quasi-static conditions.
			Energy absorption capacity increases by up to 55% in the high strain rate region.
Taherishargh *et al.* [117]	Matrix: A356 alloy Particles: Pumice, size range: 2.8–4 mm	Compression Quasi-static (3 mm·min^−1^)	Compressive strength of pumice particles is anisotropic, showing a maximum in the direction parallel to its tubular pores.
			Pumice-A356 syntactic foam is an efficient energy absorber with an average density of 1.49 g·cm^−3^, a plateau stress of 68.25 MPa, and specific energy absorption of 24.8 MJ·m^−3^.
Szlancski *et al.* [111]	Matrix: Al99.5, AlSi12, AlMgSi1 and AlCu5 alloys	Compression Quasi-static (0.01 s^−1^)	Compressive test results show plastic yielding, long and slowly ascending plateau region that ensures large EA capability.
			Matrix material and the heat treatment exert strong influences on mechanical properties of MSFs.

**Table 5:** Literature survey on iron or steel matrix syntactic foams.

Reference	Syntactic Foam Type	Testing Conditions	Results
Neville and Rabiei [82]	Matrix: low carbon steel or stainless steel Particles: HS-low carbon steel (3.7–1.4 mm) or HS-stainless steel (2 mm)	Quasi-static	EA at densification was higher for stainless steel compared to carbon steel syntactic foam.
			Maximum energy absorption at densification was 68 MJ·m^−3^ for stainless steel syntactic foam.
Castro and Nutt [83]	Matrix: steel Particles: steel or alumina	Compression at 8 × 10^−4^ s^−1^	Low carbon and medium carbon syntactic steel foams have EA capacities of 69.45 and 122.68 MJ·m^−3^, respectively.
			Increasing carbon contents enhances yield strength of steel foams.
Castro and Nutt [84]	Matrix: steel Particles: steel or alumina	Compression at 8 × 10^−4^ s^−1^	Maximum EA at densification is 104.78 MJ·m^−3^.
			Relative density of steel foam increasing enhances compressive strength and decreases plateau stress.
			EA capacity increased by six times per unit mass and 70 times per unit volume when compared to Al-foams.
Peroni *et al.* [79,80]	Matrix: 99.7% pure iron Particles: S60HS (d 30 μm) or iM30 K (d 18 μm) glass hollow particles in 5, 10 and 13 wt.%	Quasi-static (10^−2^ s^−1^) Low(10–20 s^−1^) High (1000–2000 s^−1^)	Yield strength increases with strain rate, being 47% higher in comparison to that measured under quasi-static conditions.
			Increasing glass microspheres contents reduce the strength of MSFs.
			Strength and fracture behavior of MSFs depend on the intrinsic properties of glass microspheres used.
Weise *et al.* [89]	Matrix: FeNi36 Particles: S60HS (d 30 μm) glass powders	Tension	Using fine powders is beneficial for mechanical properties.
			Lowering density by 30% implies a 60% reduction in ultimate tensile strength.
			Limited ductility retained under tensile load even for small additions of glass S60HS.
Weise *et al.* [85]	Matrix: AI 316L Particles: S60HS (d 30 μm) glass hollow particles at 5.3 and 10 vol.%.	Compression, tension	High sintering temperatures lead to disintegration of glass microspheres, porosity retained, but glass phase embedded within the metal phase rather than supporting pores as in a true syntactic foam.
			Property scaling of QS compressive strength according to a power law with exponent 1.13, in between typical values for syntactic and non-syntactic closed-cell foams.
Peroni *et al.* [86]	Matrix: AISI 316L Particles: glass microspheres S60HS (d 30 μm) glass hollow particles at 40 and 60 vol.% Fillite 106 cenospheres at 40 vol.%	Compression Quasi-static (10^−2^ s^−1^) Low(10–20 s^−1^) High (1000–2000 s^−1^)	Cenospheres remain intact and yield high quality syntactic foam.
			Strength loss with decreasing density less significant for cenosphere-compared to glass microsphere-based materials.
			Compressive strength increases by 25% for glass and cenosphere-based variants with strain rate, a dependence that is attributed to lattice structure.
Brown *et al.* [87]	Matrix: low carbon steel or stainless steel Particles: low carbon steel or stainless steel	Three-point bending	Flexural yield strength of 40 MPa, which is close to the compressive yield strength (42 MPa).
			Plateau strength under compression is 50% higher than ultimate bending strength.
			Ductile failure due to propagation of pre-existing microporosity in the matrix.
Vendra *et al.* [88]	Matrix: low carbon steel or stainless Steel Particles: low carbon steel or stainless steel	Compression– compression fatigue	After 1 million cycles at fatigue load of 50% of the maximum plateau strength, stainless steel MSFs show a total strain of 8%.
			Superior fatigue properties due to strong bonding between the hollow spheres and matrix.
Luong *et al.* [90]	Matrix: iron or FeNi36 Invar Particles: hollow glass microballoons (GMB)—5 and 10 wt.%	Compression Quasi-static 10^−3^ s^−1^ High (strain rates up to 2500 s^−1^)	Yield strength decreases with increasing GMB content.
			Quasi-static yield strengths of iron MSFs containing 5 and 10 wt.% GMB are 14% and 17% lower than that of the matrix alone.
			Yield strength of Invar MSFs containing 5 and 10 wt.% GMB are 35% and 51% lower than that of the matrix alone. However, specific strength increases with GMB content and exceeds the respective data of other iron and steel foams.

The properties of MSFs produced by different methods and from different materials have been compared in several works and confronted with those of the conventional Al-foams [74]. From the published works, it was demonstrated that the stress-strain compressive curves of MSFs strongly depend on the material constituting the hollow spheres. The compressive strength of these MSFs is controlled by the strengths of metal matrix and of the hollow spheres. The volume fraction, structure and distribution of the hollow spheres exert considerable effects on the properties of MSFs.

**Figure 6:**
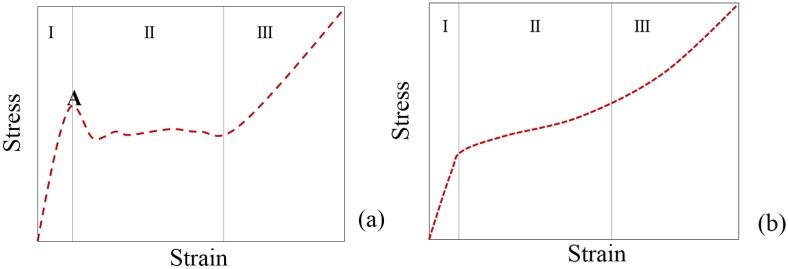
Representative quasi-static compressive stress-strain graphs (**a**,**b**) of metal matrix syntactic foams showing the linear elastic region (I), plateau region (II) and the densification strain (III).

There are two types of the stress-strain curves (Figure 6). In general, MSFs containing ceramic hollow spheres, such as alumina and silicon carbide show three main regions (Figure 6a): (I) linear elastic deformation where the stress increases linearly with strain until reach a peak stress; (II) stress drops and is followed by a plateau; (III) densification. The strong and brittle ceramic hollow spheres fail before metallic matrix being responsible by the first stress drop. Other MSFs, in particular the MSFs containing metal hollow spheres the stress-strain curves are characterized by the same three regions (Figure 6b), but the yield stress point do not have a well-defined yield point.

For example, the mechanical properties (axial compressive and plateau strengths) of MSFs based on Al-alloy A356 were higher (160 MPa, [94]) when reinforced with hollow SiC spheres than when using fly ash cenospheres (75 MPa) [110]. It was also shown that the plateau stress and the energy absorption capability of MSFs were strongly dependent of the volume fraction of reinforcing material, with the mechanical properties of ceramic particles having a minor impact on the properties of bulk material.

Recently, MSFs reinforced with several kinds of ceramic microspheres and by-products have drawn a lot of attention due to their low density, heat insulating, saving energy and high mechanical features [98]. Millions tons of ceramic microsphere powders are generated in coal based thermal power plants every year and only a small portion is being utilized. Metal matrix MSFs reinforced with ceramic microspheres proved to possess better compressive properties than conventional Al-foams. The uniform distribution of ceramic microspheres in the metallic matrix MSFs prepared by melt-foaming method, the thicker pore walls and their generally higher density in comparison to conventional Al-foams are factors that contribute to the enhanced mechanical performance of MSFs.

Expanded perlite was successfully attempted as alternative reinforcement to prepare Al-alloy MSFs. Such achievement combined with the relatively low production costs of MSFs by infiltration casting stimulated the production of high amounts of expanded perlite particles that further contributed to decrease the production costs of MSFs. A few available literature reports [99,100,101] revealed that mechanical properties of perlite-MSFs increase by decreasing particle size of reinforcement and by post heat treatment. Further research is needed. The properties of MSFs have been tested for evaluating their suitability for commercial and military applications [118,119,120]. Rabiei and Vendra compared different MSFs based on Al-alloys and steel alloys with conventional Al-alloy foams (Table 6).

**Table 6:** Properties of composite metal foams with conventional metal foams [114].

Sample Property	MSFs				Hollow Sphere	Conventional Foams	
	PM Carbon Steel	PM SS Foam	Al-LC Cast Foam	Al-SS Cast Foam	SS HSF	Al-Foam	Al-Foam
Sphere OD (mm)	3.7	2.0	3.7	3.7	2–3	3	3
Wall thickness (mm)	0.2	0.1	0.2	0.2	0.25	–	–
Density (g·cm^−3^)	3.06	2.95	2.43	2.45	1.4	0.4	0.24
Relative density (%)	38.9	37.5	42.5	42.5	17.8	14.8	8.9
Plateau Stress (MPa)	36.2	127	60	105	23	5	2.5
Densification strain (%)	54	54	57	57	60	68	50
Strength/density	12	43.7	24.4	43	16	12.5	10
Energy absorption at densification (MJ·m^−3^)	18.9	67.8	31	51	13	2.6	1.32

In summary, compressive strength and EA efficiency of MSFs is considerably higher than the conventional closed-cell metal foams [114]. The differences relay on the higher densities and thicker cell walls of MSFs. The high weight and the high volume of MSFs, compared to the conventional foams, are the main limiting factors for their widespread structural applications.

Table 7 summarizes the data reported for the different fillers used to prepare MSFs based on Al-alloys. The following general trends are apparent: (i) stronger alloys originate stronger MSFs; (ii) Smaller size fillers favor the strength of MSFs; (iii) proper heat treatments enhance the mechanical properties of MSFs.

**Table 7:** Literature data on other aluminum alloy syntactic foams with different fillers.

Matrix	Filler Material	Filler Size	Filler Particle Density (g·cm^−3^)	MSFs Density (g·cm^−3^)	Plateau Stress (MPa)	References
Pure Al	Cenosphere	90–150 μm	1.00–1.74	1.52–1.43	63–42	Wu *et al.* [121]
A356	Cenosphere	45–125 μm	0.7	1.25–2.1	45–180	Rohatgi *et al.* [122]
Pure Al	Ceramic HS: 45 SiO_2_-35 Al_2_O_3_-20 Mullite	100–1450 μm	0.57–0.81	1.43–1.49	77	Orbulov and Ginsztler [123]
Pure Al	Ceramic HS: 60 SiO_2_-40 Al_2_O_3_-20 Mullite	250–500 μm	0.75	1.38	62	Zhang and Zhao [124]
Pure Al	Ceramic HS: 60 SiO_2_-40 Al_2_O_3_-20 Mullite	75–125 μm	0.6	1.45	92	Tao and Zhao [109]
A356	Ceramic HS: SiC	1 mm	1.160	1.819	110	Luong et. al [94]
A356	Ceramic HS: Alumina	3 mm	–	1.6–2.11	62.8	Licitra *et al.* [93]
Pure Al	Glass HS: 60 SiO_2_-40 Al_2_O_3_-15 CaO-Na_2_O	0.5–4 mm	0.95–0.65	1.58–1.88	42	Zhang and Zhao [124]
A35	Expanded Perlite	3–4 mm	0.18	1.05	45	Fiedler *et al.* [100]
A356	Pumice	2.8–4 mm	0.76–0.80	1.48–1.50	64–76	Taherishargh *et al.* [117]
Al 99.5	Iron (Fe pure) HS	1.92 ± 0.07 mm	0.093	1.41	35–39	Szlancski *et al.* [111]
AlSi12	Iron (Fe pure) HS	1.92 ± 0.07 mm	0.093	1.42	55–61	Szlancski *et al.* [111]
AlMgSi1	Iron (Fe pure) HS	1.92 ± 0.07 mm	0.093	1.60	54–70	Szlancski *et al.* [111]
AlMgSi1-T6	Iron (Fe pure) HS	1.92 ± 0.07 mm	0.093	1.60	75–91	Szlancski *et al.* [111]
AlCu5	Iron (Fe pure) HS	1.92 ± 0.07 mm	0.093	1.72	47–101	Szlancski *et al.* [111]
AlCu5-T6	Iron (Fe pure) HS	1.92 ± 0.07 mm	0.093	1.72	120–162	Szlancski *et al.* [111]

## 3. Nanocomposite Metal Foams

One strategy to improve the mechanical properties of conventional foams is using nano-sized reinforcements (e.g., particles, fibers, nanotubes) instead the micro-sized reinforcements. Foams reinforced with nano-sized reinforcements are called nanocomposite metal foams. The nanoscale reinforcements are much more effective in improving the desired properties in comparison to the microscale reinforcements due to their high interface-to-volume ratio. Some advantages of using nanoscale reinforcements are the much smaller volume fractions required and the negligible weight contributions for the resulting nanocomposite metal foams.

### 3.1. Metal Foams Reinforced with Ceramic Nanoparticles

The addition of nano-sized ceramic (e.g., alumina [125] and SiC [126]) particles was shown to enhance foam stability and homogeneity of the cellular structure without causing structural defects. Different *ex-situ* and *in-situ* strategies have been developed to uniformly disperse the nanoparticles in the powders mixture using ultrasonic methods, or incorporate them in molten metal through an *in situ* reaction. After the first screening results, several works have been conducted to study the effects of nanoparticles on the mechanical performance of the resulting nanocomposite foams prepared by using both direct and indirect foaming methods. For example, Al-foams reinforced with SiC nano-particles (nano-SiC_P_/Al composite foams) were prepared by mixing nanoparticles within aluminum powders using high-energy ball milling. After that, calcium carbonate (as blowing agent) was added to the initial mixture using a mechanical mixer. A dense foamable precursor material was prepared by hot pressing the mixture containing all the solids and heated at temperatures close to melting temperature to obtain the reinforced foams. The results have shown that nano-sized ceramic particles reduce the brittleness of the foams in comparison to micro-sized ones. Moreover, small additions of nanoparticles improved the foam structure, refined pore size and homogeneity of the pore distribution leading to significant enhancements of yield stress (194.5%), plateau stress, an increase of energy absorption (69.4%) in comparison to the conventional aluminum foams [126]. The pores of nanocomposites foams were much finer than those of pure Al-foams, changing from millimeter to micrometer range. In the reality, nanoparticles act as stabilizers and reinforcement agents.

Nanoscale reinforcements have been incorporated in several types of matrices to obtain nanocomposite foams, but the number of available literature reports is scarce [126,127,128,129]. A small addition (usually less than 2 wt.%) of nanoparticles can significantly strengthen the metal matrix, solving the problems of current Al-foams incorporating high loadings (generally above 10 vol.%) of microscale reinforcements required to achieve the desired levels for the mechanical properties (e.g., elastic modulus) and dimensional stability, thus compromising the weight and the toughness of the final composites.

The main difficulties to overcome in producing nanocomposites are the high surface to volume ratio and the generally low wettability of ceramic particles by aluminum. Smaller particles have a stronger tendency to agglomerate and to form micrometric clusters, losing their effectiveness in obstructing the movement of dislocations. For this reason, they cannot be prepared by simple conventional manufacturing processes. It is crucial to modify these methods.

### 3.2. Metal Foams Reinforced by Metal Deposition

Casting, powder metallurgy (slurry foaming and loose powder) and metallic deposition are the main manufacturing processes to fabricate these open-cell foams. An approach to improve the conventional open-cell Al-foams involves the electrodeposition into the cellular structure of metals having superior mechanical properties (e.g., nickel and copper). The aims are increasing the plateau stress, delaying the initial densification strain, and increase the energy absorption capacity. Few papers have been published in this field [130,131,132,133,134,135,136,137]. The first paper on this topic published in 2008 [130] reported on the effects of Ni-W coatings on the mechanical properties of Al-foams. The mechanical properties of the original open-cell foams were enhanced by increasing the coating thickness (density). The structure could be tailored and the maximum specific stress was substantially improved by coating with nickel. Jung *et al.* [137] reported considerable increases in plateau stress of an Al-alloy foam reinforced with nano-crystalline nickel deposition (Ni coating thickness ~250 nm). They also found that thick coatings considerably reduced the densification strain, thus compromising the energy absorption capacity of foams. Manipulating the deposition parameters (currents and times) allowed adjusting the extent of deposition. The energy absorption capacity was directly proportional to density or deposition time. However, uniform coatings could hardly be achieved for shortest deposition time while the longest one did not brought further benefits when compared to the intermediate deposition times. Such coatings also improved the resistance to corrosion which then allows using these foams even in aggressive environments. This strategy can broaden the functional applications of open-cell metallic foams, for example, as heat exchangers due to their very high thermal conductivity. The improved mechanical properties would allow producing more compact heat exchangers by partially merging the structural and heat exchanging functions.

Copper electro-deposition has been also considered to reinforce the open-cell foams (copper electrodeposited Al-foam, 10–40 PPI) [132]. Although stiffness of copper (123 GPa) is less than that of nickel (206 GPa), the first is less expensive and has a Young’s modulus almost twice that of aluminum (69 GPa) [1]. The results of quasi-static systematic compression tests of nano-Cu coated open-cell Al-foams revealed enhanced energy absorption capability and plateau stress without compromising the densification strain. The overall mechanical performance was shown to be strongly affected by the foam relative density, cell topology (pore size, strut thickness, *etc.*) and the electro-deposition conditions. For example, the energy absorption capacity of Al-foams coated with a 60 nm Cu coating was 3 times greater than that of plain foams. The compressive stress–strain response of the composite samples showed no significant reduction of the densification strain compared to the uncoated foams due to the small change in the foam strut thickness and pore size. A comparison between thin Cu-coated and uncoated Al-foam samples with the same overall strut thickness (*i.e.*, same effective volume density and porosity) showed that the nano-reinforced foams had superior energy absorption capacity compared to the non-coated foams.

### 3.3. Metal Foams Reinforced with Carbon Nanotubes

**Figure 7:**
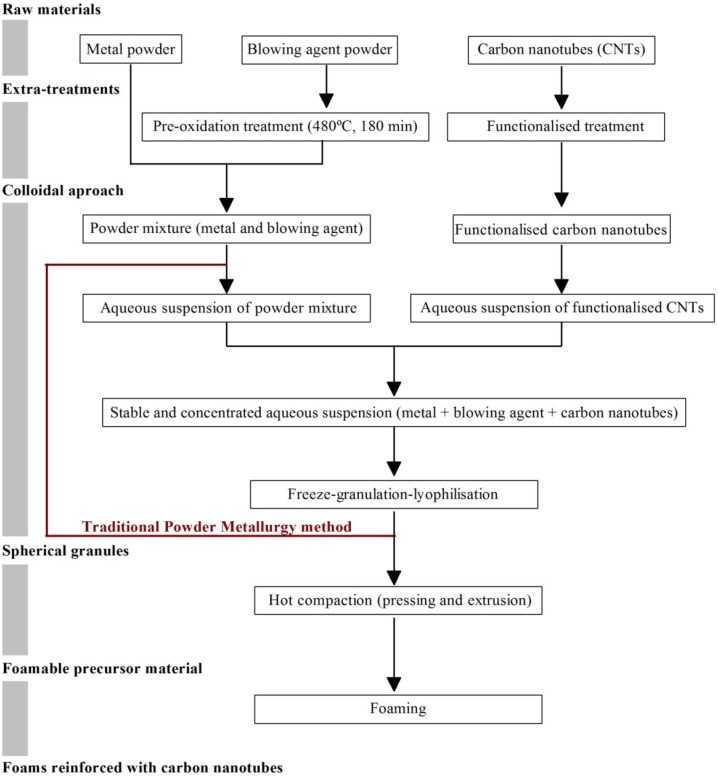
Schematics of the novel approach for preparing metal-foams reinforced with carbon nanotubes (CNTs) by combining the powder-metallurgy with colloidal-processing [138,139].

Carbon nanotubes (CNTs) have emerged as potentially ideal nano-sized reinforcements to fabricate light weight and high-strength metal-matrix composites due to their low density and high values of aspect ratio, mechanical strength, electrical and thermal conductivities. However, the incorporation of CNTs into the metal-matrices is not trivial because of their high tendency to form clusters, poor dispersion ability, and poor wettability of carbon by molten metal (due to a large difference of surface tensions). The formation of interfacial reaction products in molten metals is another limitation to their widespread use as reinforcements. Various processing strategies (e.g., powder metallurgy, molecular-level mixing, plasma spraying and casting) have been employed to overcome these problems, but with limited success [20]. The achievement of uniform dispersion of CNTs in the metal-matrix, the formation of strong interfacial bonding, and the retention of structural integrity of CNTs are the main challenges to overcome for successfully developing metal-matrix nanocomposites for industrial applications. These are key requirements to potentiate the homogeneous 3D reinforcing role of CNTs and to provide an efficient load transfer. As a matter of fact, CNTs in the generality of the literature reports on metallic matrix tend to appear in clusters, therefore, annulling their reinforcing potential. The research in this field is still in its infancy with only three articles published in 2015 [138,139,140,141]. The first articles in the field were published by Duarte *et al.* [138,139] and disclose a novel approach combining colloidal-processing (including freeze-granulation-lyophilisation) and powder PM method as schematized in Figure 7.

**Figure 8:**
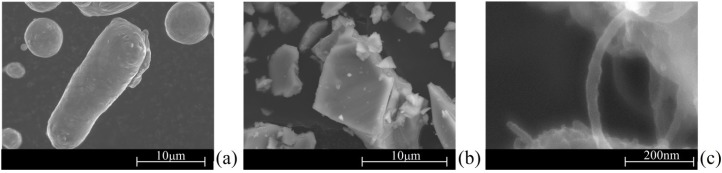
SEM micrographs of the starting raw materials: (**a**) Al-12 alloy; (**b**) TiH_2_ powder; (**c**) multiwall carbon nanotubes (MWCNTS)-COOH.

Figure 8 shows Al-alloy powder Al-12Si), titanium hydride powder (TiH2, as blowing agent) and –COOH-functionalized multiwall carbon nanotubes (MWCNTs-COOH, as reinforcement elements) which were the main starting raw materials used in this work.

The first paper [138] reports preliminary results on the preparation of spherical granules by free granulation and preparation of precursor materials, showing evidences of the good dispersion of the MWCNTs inside the precursor materials. The second one [139] discloses the effects of the most relevant processing steps in a more systematic way, including a detailed characterization of the starting raw materials and the pre-oxidation heat treatment on the foaming agent. A detailed study of the dispersion behavior of the MWCNTs using different dispersing agents and their synergetic dispersing actions was preformed through complementary techniques (sedimentation and zeta potential measurements using diluted suspensions and rheological measurements using concentrated suspensions).

**Figure 9:**
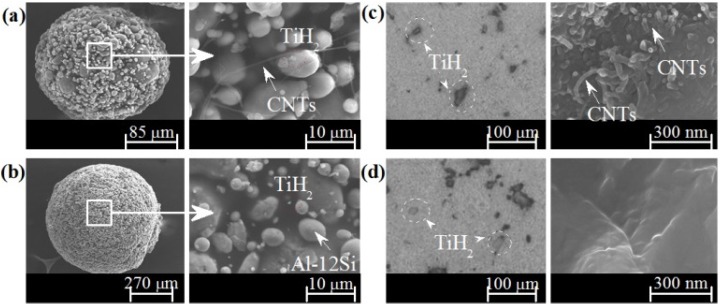
Highly-spherical granules prepared by freeze-granulation-lyophilisation and its surface with (**a**), and without (**b**), MWCNTs-COOH. Microstructural features of precursor materials (and of their fracture surfaces) prepared from granules with (**c**), and without (**d**), MWCNTs-COOH.

The SEM images in Figure 9 show that highly-spherical granules were obtained irrespective of the absence (Figure 9a) or the presence (Figure 9b) of MWCNTs-COOH. These SEM images also provided clear evidences about the uniform dispersion of MWCNTs-COOH and TiH_2_ in the lyophilized granules (Figure 9a,b) and in the precursor materials (Figure 9c,d).

Figure 9c reveals a random distribution of individually dispersed and stretched MWCNTs-COOH aligned perpendicular direction of the hot compaction, remaining its structural integrity. This contrasts with the complete absence of MWCNTs-COOH in the non-reinforced sample (Figure 9d), as expected. As shown in Figure 9a, MWCNTs-COOH appear individually dispersed and stretched avoiding their natural tendency to form clusters that annul their reinforcing potential. Furthermore, the TiH_2_ are also uniformly dispersed (dashed-circled, Figure 9a,b) ensuring the foam quality. The key for the successful preparation of Al-alloy foams reinforced with MWCNTs is ensuring a good dispersion of the MWCNTs. These highly-spherical granules were easily hot compacted to form dense precursor materials showing typical microstructures comparable to those of precursors prepared by the PM method as seen in Figure 9c and Figure 9d for precursors with and without MWCNTs-COOH, respectively.

**Figure 10:**
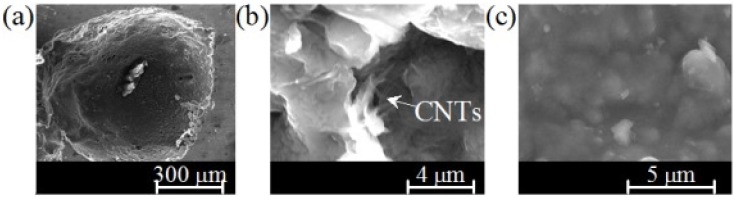
Cellular-pore (**a**) and magnified images of its pore-wall microstructure with (**b**) and without MWCNTs-COOH dispersed in its aluminum-matrix (**c**).

The novel approach, a modification of the traditional powder metallurgy (PM) method by adding an advanced colloidal processing step allows: (i) achieving an effective dispersion of the functionalized MWCNTs-COOH in aqueous media and the homogeneous mixing with the other components of the system; (ii) preserving the homogeneity and structural integrity (tubular structure) of the carbon nanotubes through the process; (iii) establishing strong bonds between MWCNTs and metal matrix, which provide an efficient load transfer. Accordingly, in comparison to the non-reinforced Al-foams, the mean values of Vickers micro-hardness of reinforced ones increased within the range from 55% to 125%, depending on the neighborhood between of the indentation point and the reinforcing MWCNTs. However, further research efforts will be required to systematically investigate the effects of other relevant experimental variables not yet covered such as: (i) the reproducibility and overall quality control of the foaming process; (ii) determining the maximum allowable content MWCTs that can be incorporated without degrading the foaming process or the quality of the foams; (iii) applying the method to other Al-alloys and metallic systems; (iv) evaluating the properties and performance of the foams in high-tech applications and demonstrate their unique and competitive advantages over their existing counterparts. Individualized and stretched MWCNTs can be seen randomly distributed in the aluminum-matrix (Figure 10). This non-agglomerated condition confirms the effectiveness of the dispersion achieved in the colloidal processing step. The stretched condition is likely to be boosted upon foaming, being favorable to reinforcement.

The Vickers microhardness measured on the struts (cell-walls) changed from (60 HV ± 5.18 HV) for non-reinforced Al-foam to 93.43 ± 19.30 HV for the sample containing 0.5 wt.% MWCNTs, with some values exceeding more than double of the non-reinforced ones. The high standard deviation of reinforced foams could be attributed to: (i) the small added amount of MWCNTs; (ii) the neighborhood between of the indentation point and the reinforcing MWCNTs. This means that hardness can randomly change from one point to another depending on the closeness vicinity of a reinforcing MWCNT.

**Figure 11:**
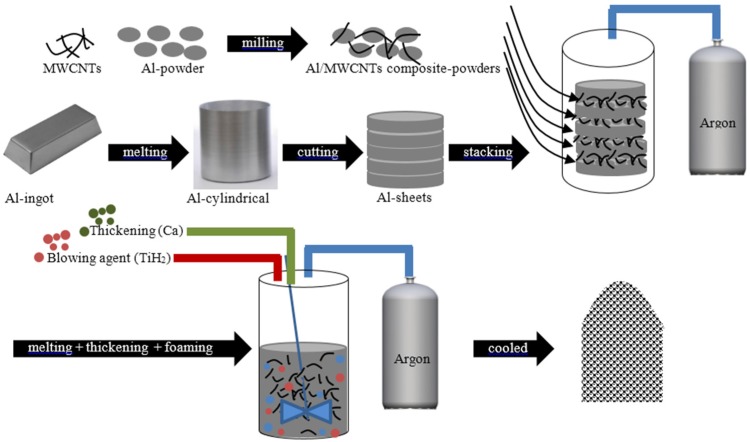
Schematics of the fabrication process of closed-cell Al-foams with and without MWCNTs [140].

Zhang *et al.* [140] developed closed-cell Al-foams reinforced with different contents of MWCNTs by using a modified melt foaming method (Figure 11). High-energy ball-milling with adjusted the milling parameters was used to disperse the MWCNTs into the powders. According to the authors, MWCNTs existed mainly in three forms: totally embedded in cell wall; partially embedded in cell wall; and totally exposed on cell wall surface. The formation of interfacial intermetallic product (e.g., Al_4_C_3_) due to the reaction between the MWCNTs and the Al-alloy was reported to occur, which allegedly would lead to strong interfacial bonding between MWCNTs and aluminum matrix. Nevertheless, high-energy mixing process (e.g., ball-milling or mechanical-alloying) are likely to cause structural damage and structural-integrity loss to CNTs. Dispersion improvements of CNTs in the Al-matrix using extended ball milling times have been reported together with some structural damages. This is particularly serious for the single-walled CNTs since their tubular integrity is lost. MWCNTs may still provide the desired structural-integrity even suffering some damage in the outer-walls. The eventual formation interfacial-products (intermetallics) due to interfacial reactions between CNTs and molten metal under harsh conditions can also lead to loss of structural-integrity.

These authors studied the effect of volume fraction of MWCNTs (0.0–1.0 wt.%) on the compressive behavior and energy absorption of these nanocomposite foams. They found that the compressive yield strength increased up to 0.5 wt.% MWCNTs, while the opposite trend was noticed with further added amounts of MWCNTs. For example, the compressive yield strength of nanocomposite foams containing 1 wt.% MWCNTs was inferior to that of non-reinforced foam (without MWCNTs). Similar conclusions about the influence of the MWCNTs were drawn for the energy absorption curves that are obtained by integrating the stress-strain curves, with the highest and the lowest energy absorption capacity values being measured for the foams with 0.5 wt.% and 1.0 wt.% of MWCNTs, respectively. Although the influence of different MWCNTs contents on the mechanical properties of composites is likely to depend on the specific materials matrix, and especially on the interfacial bonding strength between the reinforcing MWCNTs and the embedding matrix, it is important underlying that the required amounts of MWCNTs for an effective reinforcement are usually small (~0.5 wt.%), provided that they are well dispersed. The probable non-fulfilment of this condition in work reported by Zhang *et al.* [140], especially for MWCNTs contents > 0.5 wt.% would explain why energy absorption capability the resulting Al-foams was even worse than for non-reinforced ones.

**Figure 12:**
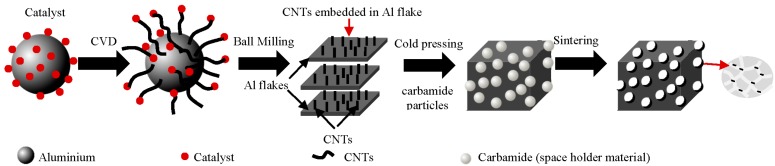
Schematic diagram of the procedures used to fabricate CNT/Al [141].

A method for *in-situ* growing carbon nanotubes (CNTs) to reinforce Al-foams derived thereof was proposed by Wang *et al.* [141]. The method, combining chemical vapor deposition (CVD), ball milling and space-holder technique, is schematized in Figure 12. The CNTs were grown onto the surface of Al-alloy particles by *in-situ* chemical vapor deposition and the Al/CNTs powders were then used to fabricate the nanocomposites. The authors claim that the proposed method allows CNTs to be well dispersed and integrated in Al powders and fosters good interfacial bonding between CNTs and Al matrix, while morphology and size of pores could be well controlled by the space-holder technique.

The SEM images of the resulting nanocomposite foams show some intercommunicating pores derived from the mutual contacts among the carbamide spheres, but also some closed pores. The type of foams obtained (closed cell *vs.* open cell) strongly depends on the added volume fraction of the pore generating agent. Below the percolation threshold, the pores tend to appear separated from each other and closed cell foams are formed. From this perspective, the intercommunicating voids could even be considered as structural imperfections. The authors [141] also investigated the effects of ball milling on the compressive behavior and energy absorption capacity of non-ball-milled and ball-milled composite foams prepared by compacting the Al/CNTs powders without or after being ball milled, respectively. The properties of the resulting nanocomposite foams were compared to the non-reinforced Al-foams (without CNTs) obtained using the conventional space-holder technique. The stress-strain and energy absorption capacity curves reveal that the nanocomposites exhibited typical stress behavior of Al-foams characterized by three regions (linear elastic, plateau, densification). It is clear that ball-milling the Al/CNTs powder is essential for profiting from this *in-situ* chemical vapor deposition approach of growing CNTs onto the surface of Al-particles, as the overall properties of Al-foams made from non-ball-milled nanocomposite powders were worse than those of non-reinforced ones. Besides the superior mechanical properties of ball-milled composite foams, their stress-strain and energy absorption capacity curves are smoother. The compressive yield strength of the ball-milled 2 wt.%-CNT/Al composite foams was reported to be 25% and 67% superior in comparison to pure Al-foams and non-ball-milled nanocomposite foams (with 2 wt.%-CNTs), respectively. The authors postulated that for CNTs to effectively act as reinforcing agents they have to be uniformly distributed and without structural damage for improving the interfacial bonding between the CNTs and Al matrix, and acting as bridges to restrict the cell wall deformations.

For taking full advantages of CNTs, it is vital to understand how they act to reinforce a composite. Fortunately, this issue has been tackled recently by [142]. The researchers used a powder metallurgy route to fabricate an Al-matrix composites reinforced with 0.6 wt.% MWCNTs produced by chemical vapor deposition and performed advanced *in-situ* tensile tests by operating the tensile stage with a CNTs/Al sample inside a FE-SEM chamber. This *in-situ* SEM approach provides a direct and easy method to investigate the mechanical behavior of MWCNTs in composites, which is essentially regulated through a load transfer strengthening mechanism. When a force is applied to the composite, the MWCNTs initially act like a bridge to suppress crack growth. As further force is applied, the outer walls of the nanotubes in contact with the Al matrix start to break. The inner walls then fracture, either breaking vertically or unpeeling to expose the next inner walls, and so on. The SEM images of the completely fractured composite surface showed clear evidences of ruptured MWCNTs.

Several strengthening mechanisms have been already proposed for MWCNTs in metal-matrix composites (MMCs) [142,143,144,145,146,147] including load transfer from matrix to the MWCNTs [146]; grain refining [144] and texture strengthening [145] by pinning effect of MWCNTs; dispersion strengthening of MWCNTs [146]; solution strengthening of carbon atoms [147]; strengthening of in-situ formed or participant carbide particles [147]; and thermal mismatch between MWCNTs and matrix [6]. However, the composite strength might be a synergetic result of several strengthening mechanisms although the specific contribute of each one is not easy to discriminate from these previous reports [142,143,144,145,146,147]. In the parallel work, Chen *et al.* [147] also examined the failure behaviors of MWCNTs (produced by chemical vapor deposition) in an Al metal matrix composite reinforced with 0.6 wt.% MWCNTs in an attempt to shed further light on this issue using the same *in-situ* tensile tests reported elsewhere [147]. The tensile sample was prepared by extrusion and machining. This *in-situ* advanced tensile testing technique enabled them concluding that the mechanical behavior of MWCNTs in composites is essentially controlled by a load transfer strengthening mechanism. There was an effective load transfer between the MWCNTs and the matrix and between the cell walls in the composites during the tensile failure.

Zhendong *et al.* [148] and Wang et.al. [149] patented an innovative method to prepare a foam metal-CNTs and metal-graphene composite materials comprising a metal foam substrate and a graphene film layer positioned on the substrate. The metal-graphene composite foam was prepared by means of electrophoresis. Specifically, the preparation method included the following steps: removing greasy dirt and oxides from the surface of the metal foam substrate, preparing graphene by the oxidation-reduction method, modifying graphene, and performing electrophoretic deposition of graphene onto the surface of the metal foam substrate. Within certain of electromagnetic waveband, the metal-graphene foam composite material has the structural advantages of light weight and porosity, large specific surface area, and good conductivity. On the other hand, the composite material integrates excellent electrical conductivity and high dielectric constant, a capacity of being more conductive to absorbing electromagnetic waves due to a large amount of defects and functional group residues and other properties of the self-made graphene. The composite material was claimed to have excellent electromagnetic shielding performance.

### 3.4. Metal Foams Reinforced with Short Fibres

Short ceramic (Al_2_O_3_) fibers are more effective than ceramic particles in enhancing the viscosity of metallic melts due to their high aspect ratio. Therefore, their use as stabilizing agents for the fabrication of metal foams has been attempted. However, only a few reports on this topic are available in open literature.

Liu *et al.* [150,151] fabricated closed-cell Zn-22Al composite foams reinforced with 3 vol.% of short Al_2_O_3_ fibers by the conventional direct foaming method melt using CaCO_3_ as blowing agent. Zn-22Al matrix was melted at 590 °C in a graphite crucible in an electric furnace, and then CaCO_3_ powder as blowing agent was then added into the melt under mechanical stirring (900 rpm) for 2 min. The temperature was increased to 700 °C–720 °C and held for several minutes to allow the release gas bubbles from the decomposition of the blowing agent. Finally, the composite foams were cooled down.

The distributions of the short fibers in the composite foams were observed by SEM and the compressive properties of the composite foams were investigated in quasi-static condition. The short Al_2_O_3_ fibers in the composite foams were mostly distributed in two locations: some uniformly dispersed in cell edges/walls; and others are penetrating through the cells. The Zn-22Al foams reinforced with short Al_2_O_3_ fibers exhibit higher compressive yield stress and energy absorption capacity than the non-reinforced Zn-22Al alloy foams. Moreover, the compressive curves of composite foams are smoother without any dentate collapse plateau region, and increase more rapidly than those of Zn-22Al alloy foams.

### 3.5. Metal Foams Reinforced with Spinels

Synthesizing or growing reinforcing nano- or sub-micron particles inside the matrix during manufacturing process through an *in situ* reaction is an alternative approach. Such *in situ* reactions can also be used to make grain refiners in melts. For example, SiO_2_ dispersed into the AlMg5 alloy melt form 3.4 vol.% of spinel particles that were sufficient for an efficient foam stabilization [152]. *In-situ* fabrication enables accomplishing homogeneous distribution of the reinforcements and their good wetting by the matrix at lower costs in comparison to the *ex-situ* method.

**Figure 13:**
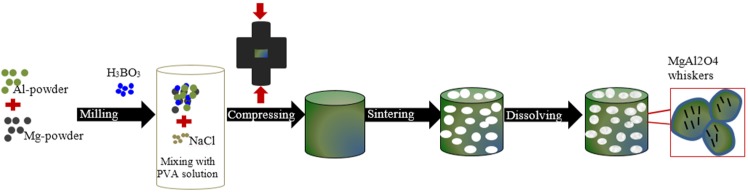
Schematic illustration of procedures to fabricate Al composite foams reinforced byMgAl_2_O_4_ spinel whiskers [153].

Guo *et al.* [153] applied to the first time a method for *in-situ* generating the reinforcing elements. MgAl_2_O_3_ nano-wiskers reinforced metallic foams were fabricated via sintering and dissolution processes using sodium chloride particles as a space holder material (Figure 13). Such spinel nano-wiskers (50–300 nm and the aspect ratio of 10–50) are ideal reinforcement for Al composite foams. The MgAl_2_O_3_ spinel whiskers are generated in the cell wall and might exists in three forms: (i) entirely embedding in the cell walls; (ii) partially protruding through the cell walls; (iii) penetrating through the micropores. The improvements brought by the *in-situ* MgAl_2_O_3_ spinel whiskers in compressive properties and energy absorption suggest the usefulness of the method to prepare Al composite foam with excellent properties.

## 4. Future Directions

The paper summarizes all important manufacturing routes of MMCs and describes the role of particles in regard of foam stability and mechanical strengthening comprising. As this topic is of great interest for industry and academia—as the number of publications verifies—this review article is indeed a needed task. Besides the summarizing efforts of the already published perceptions of other works, attempts were also made to draw some general conclusions.

The most important functional properties of foams include low density, essential for for light weight applications; specific energy absorption capacity required for crash safety applications; mechanical strength for the most demanding structures under load conditions; acoustic and heat insulation properties capacity for noise and heat management; and refractoriness for fireproof applications. A number of traditional processing approaches of composites and nanocomposite foams are based on both direct and indirect foaming methods. Each method offers several advantages and drawbacks, which need to be balanced according to the intended properties for any specific application. As in any other technological field, the final properties of foams derived from each manufacturing method are strongly dependent of the quality of raw materials, their right combination and the processing details that might require specific skills and knowhow.

There are at least two main methodological approaches for further enhancing the properties of metallic foams, depending on the intended set of final properties: The first one involves exerting a closer control over all the relevant processing steps and experimental parameters of the relatively low density foams manufactured by the traditional methods, including the search for more effective reinforcing agents (particles, carbon nanotubes, graphene, short fibers, *etc.*) and their uniform distribution in the matrix to maximize the mechanical benefits. Developing experimental methodologies for achieving uniform dispersions of the nano- and micro-sized reinforcing elements and avoiding their strong agglomeration into cluster are some of the main challenges. Achieving such targets and promoting a proper (strong) interfacial bonding to delay premature failure while avoiding interfacial reaction between the reinforcements and the metal melt and the consequent formation of undesirable interfacial products will be the future power research lines in the field. Obtaining uniform dispersion of the nano-sized reinforcing elements is perhaps the biggest challenge. Efforts should be made towards developing reinforced foams exhibiting long flat plateau, having higher yield stresses (close to the stress plateau), and possessing higher specific energy absorbing capacities. The second approach is to further explore the new concept of syntactic foams, especially when the light weight aspect can be sacrificed to favor high demanded mechanical properties. Syntactic foams are less porous but enable a close control of porosity fraction and its spatial distribution, and consequently obtain superior mechanical properties when compared to conventional metallic foams. In this last case, mass production still requires the search for suitable fillers (hollow spheres or porous particles) that should be available at an economically acceptable cost. However, the minimum achievable density and cost of MSFs is limited by the filler material. Reinforcing the metallic matrix of MSFs with nano- and micro-sized reinforcing elements would certainly be a very interesting direction to follow taking advantages of the specificities of the two main approaches described above. In all kinds of foams, a better level of understanding of the strengthening mechanisms is needed to make supported progresses in the field.
